# The P2X7 receptor modulates immune cells infiltration, ectonucleotidases expression and extracellular ATP levels in the tumor microenvironment

**DOI:** 10.1038/s41388-019-0684-y

**Published:** 2019-01-17

**Authors:** Elena De Marchi, Elisa Orioli, Anna Pegoraro, Sabina Sangaletti, Paola Portararo, Antonio Curti, Mario Paolo Colombo, Francesco Di Virgilio, Elena Adinolfi

**Affiliations:** 1 Department of Morphology, Surgery and Experimental Medicine, Section of Pathology, Oncology and Experimental Biology, University of Ferrara, Via Luigi Borsari, 46, 44121 Ferrara, Italy; 2 Department of Experimental Oncology, Molecular Immunology Unit, Istituto Nazionale dei Tumori (IRCCS), Via Amadeo, 42, 20133 Milan, Italy; 3 Department of Experimental, Diagnostic and Specialty Medicine, Institute of Hematology L. and A. Seràgnoli, S. Orsola-Malpighi Hospital, University of Bologna, via Massarenti, 9, 40138 Bologna, Italy

**Keywords:** Tumour immunology, Mechanisms of disease, Cell death and immune response

## Abstract

In the tumor microenvironment (TME) ATP and its receptor P2X7 exert a pivotal influence on cancer growth and tumor–host interactions. Here we analyzed the different effect of P2X7 genetic deficiency versus its antagonism on response against P2X7-expressing implanted tumors. We focused on immune cell expression of ATP degrading enzymes CD39 and CD73 and in vivo measured TME’s ATP. The immune infiltrate of tumors growing in P2X7 null mice shows a decrease in CD8^+^ cells and an increased number of Tregs, overexpressing the fitness markers OX40, PD-1, and CD73. A similar Treg phenotype is also present in the spleen of tumor-bearing P2X7 null mice and it is paralleled by a decrease in proinflammatory cytokines and an increase in TGF-β. Differently, systemic administration of the P2X7 blocker A740003 in wild-type mice left unaltered the number of tumor-infiltrating CD8^+^ and Treg lymphocytes but increased CD4^+^ effector cells and decreased their expression of CD39 and CD73. P2X7 blockade did not affect spleen immune cell composition or ectonucleotidase expression but increased circulating INF-γ. Augmented CD73 in P2X7 null mice was mirrored by a decrease in TME ATP concentration and nucleotide reduced secretion from immune cells. On the contrary, TME ATP levels remained unaltered upon P2X7 antagonism, owing to release of ATP from cancerous cells and diminished ectonucleotidase expression by CD4^+^ and dendritic cells. These data point at P2X7 receptor as a key determinant of TME composition due to its combined action on immune cell infiltrate, ectonucleotidases, and ATP release.

## Introduction

Molecules released in the tumor microenvironment (TME), whether by tumor or host cells, are crucial regulators of cancer growth and progression [1]. Extracellular ATP is one of the main components of the TME, where, either directly or following degradation to adenosine, affects tumor proliferation and interactions with immune cells, vascular endothelia, and surrounding stroma [2–4]. As high energy intracellular intermediate and precursor of nucleic acid synthesis, ATP is a key molecule in intracellular reactions and it is, therefore, stored at high concentration (5−10 mM) in most cells. ATP can be discharged into the extracellular space following plasma membrane disruption and via membrane transporters (ABC cassettes, pannexins, P2X7) or vesicle-mediated release [5]. Once in the extracellular space ATP acts as a potent damage-associated molecular pattern (DAMP) and accumulates at inflammatory sites [6]. Moreover, it can act as a source of the immunosuppressant adenosine via CD39 and CD73 ectonucleotidases mediated hydrolysis [3]. In vivo detection of extracellular ATP, tumor-bearing mice included, was made possible by the introduction of pmeLUC, a plasma membrane-targeted luciferase that allows real-time measurement of peri-cellular ATP [7–10]. Use of this probe allowed not only to visualize ATP within the TME but also to correlate its changes to therapeutic intervention such as chemotherapy or administration of caloric restriction mimetics [8, 11–13]. Among receptors for ATP, the best characterized for its role in cancer is P2X7 [3, 4]. The P2X7 receptor is an ATP-gated ion channel, coupled to activation of multiple inflammatory pathways including the NLRP3 inflammasome and release of cytokines such as IL-1β and IL-18 from innate immune cells [14–17]. Therefore, it is mainly through P2X7 that ATP triggers inflammation as DAMP. P2X7 is overexpressed in a wide variety of tumors and leukemias, where it is associated with cancer progression and poor prognosis [4, 18]. Moreover, P2X7 exerts a trophic activity on cell energy metabolism influencing the Ca^2+^ content of mitochondria and endoplasmic reticulum [19–21], enhancing oxidative phosphorylation and glycolysis and, therefore, increasing the overall intracellular ATP content, and stimulating growth-promoting pathways, including the PI3K/AKT and HIF1α/VEGF axes [22–24]. Accordingly, P2X7 blockade reduces cancer growth in various experimental tumor models including colon and pancreatic carcinoma, melanoma, neuroblastoma, and mesothelioma [22, 24–28]. However, and rather surprisingly, tumor growth and dissemination are increased in P2X7 null mice [26, 29]. This apparent paradox is due to reduced immune cell infiltration in tumors growing in the P2X7 null host compared to their wild-type (WT) counterpart, suggesting that lack of P2X7 hampers immune cell detection of tumor antigens and/or migration. Need of immune cell P2X7 expression for an efficient anti-tumor response was confirmed by bone-marrow transplant experiments showing that hematopoietic transfer from P2X7 WT into P2X7 null mice restored a near normal anti-tumor response [26]. Therefore, this study was planned to investigate the mechanism by which P2X7 genetic deletion or pharmacological blockade modulate tumor infiltration by immune cells. In the P2X7 null host, an immunosuppressive infiltrate, characterized by fewer CD8^+^ and an increased number of Treg cells, predominates. Furthermore, Treg cells express high levels of the fitness markers OX40, PD-1, and CD73. On the contrary, P2X7 pharmacological blockade in the P2X7 WT host supports a tumor-aggressive infiltrate characterized by a high number of CD4^+^ effector T lymphocytes (Teff), reduced expression of OX40 on Tregs and of CD39 and CD73 on Teff, and conventional dendritic cells (cDCs). Genetic deletion versus pharmacological blockade of the P2X7 receptor also had a differential effect on the ATP content of the TME, as while in the former case we observed a large reduction in the ATP concentration, no changes were observed in the latter. Our findings show that P2X7 receptor is a crucial determinant of tumor–host interaction since its expression and function affect both immune cell infiltration and ATP content of the TME. P2X7 pharmacological blockade does not replicate the immunosuppressive effects due to genetic ablation, rather it enhances tumor infiltration by CD4^+^ T effector cells and diminishes CD39 and CD73 expression, thus reducing immunosuppression in the TME. Our observations support the hypothesis that administration of P2X7 antagonists may be a viable therapy for cancer, combining the direct inhibitory effect on tumor growth with the promotion of a tumor-aggressive immune infiltrate.

## Results

### Tumors growing in P2X7 null mice show an immunosuppressive microenvironment characterized by increased Tregs overexpressing OX40, PD-1, and CD73

**Fig. 1:**
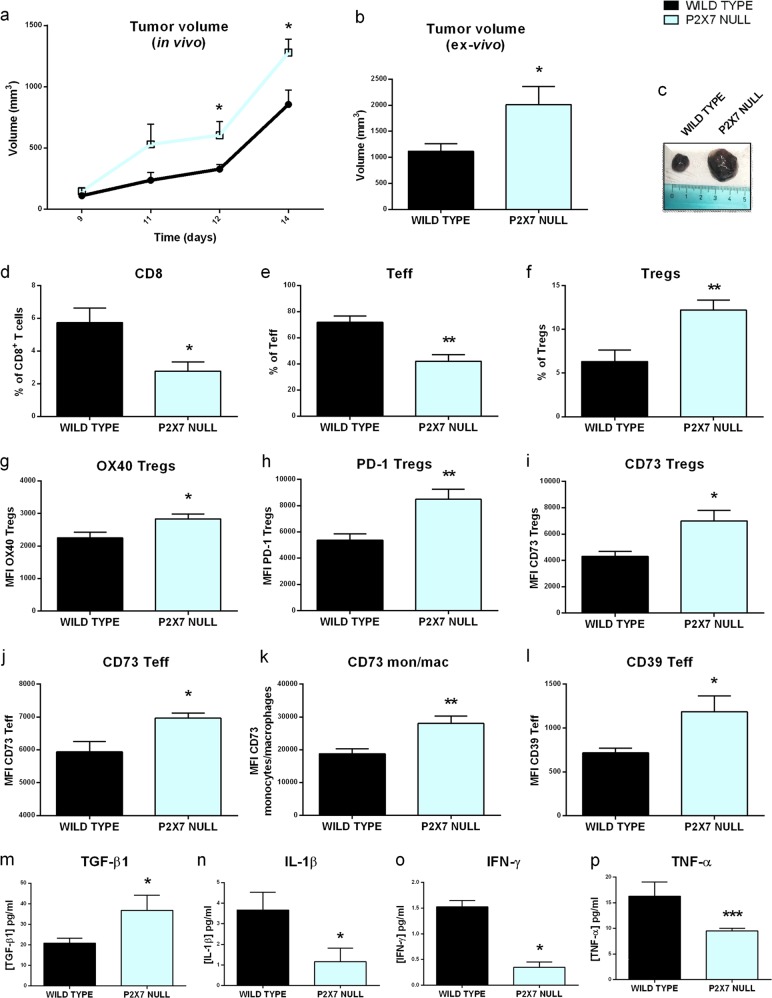
P2X7 loss enriches tumor infiltrate in Tregs overexpressing OX40, PD-1, and CD73 and decreases proinflammatory cytokines. **a–c** C57bl/6 mice were inoculated into the right hind flank with B16-pmeLUC cells in WT and P2X7 null mice. **a** Tumor volume was in vivo assessed at the indicated time points, **b** ex vivo tumor volume assessed by a calliper, and **c** representative pictures of tumors from WT and P2X7 null mice at post-inoculum day 14. Data are shown as the mean ± SEM (WT, *n* = 6–12; P2X7 null, *n* = 5–12). **d–l** Flow cytometric analysis of tumor masses from C57bl/6 WT and P2X7 null mice. **d** Percentage of CD8^+^ infiltrating T cells, **e** percentage of Teff (CD25, Foxp3, CD4^+^ gate), **f** percentage of Tregs (CD25^+^, Foxp3^+^, CD4^+^ gate), **g–i** mean fluorescence intensity (MFI) of OX40 (**g**), PD-1(**h**), CD73 (**i**) on Tregs, **j** MFI of CD73 on Teff, **k** MFI of CD73 on monocytes/macrophages gate (CD11b^+^Ly6C^+^ F4/80^+^), **l** MFI of CD39 on CD4^+^, CD25^−^ Teff. Data are shown as the mean ± SEM (WT, *n* = 4–9; P2X7 null, *n* = 4–9). **m–p** Levels of plasma cytokines of tumor-bearing C57/bl6 mice inoculated with B16-pmeLUC cells. TGF-β1 (**m**), IL-1β (**n**), IFN-γ (**o**), and TNF-α (**p**) were evaluated in plasma samples obtained at post-inoculum day 14. Data are shown as the mean ± SEM (WT, *n* = 5–14; P2X7 null, *n* = 5–11). *P < 0.05, **P < 0.01, and ***P < 0.001

Although we recently demonstrated that impaired immune infiltration accelerates tumor growth in P2X7 null mice [26], the immune contexture of tumors growing into P2X7-deficient host remained uncharacterized. Therefore, we are now providing the immuno-cytometric analysis of main tumor-infiltrating lymphocytes and myeloid cell populations in P2X7 WT and *p2x7*^−/−^ tumor-bearing mice (Fig. 1). Tumors generated by injection of murine B16 melanoma cells constitutively express high levels of P2X7 regardless the P2X7 host genotype [26]. Melanoma growth is accelerated in the P2X7 null host (Fig. 1a–c), in correlation with a significant decrease in the CD8^+^ and CD4^+^ effector T cells (Teff, CD4^+^, CD25^−^) infiltrate (Fig. 1d, e). The infiltrate is also characterized by a doubling of the Treg population (CD4^+^, CD25^+^, Foxp3^+^; Fig. 1f), which up-regulated the fitness markers OX40 and PD-1 (Fig. 1g, h). Notably, the ectonucleotidase CD73, which is often overexpressed in cancer-associated Tregs [30], was significantly increased in P2X7 null mice not only on Tregs (Fig. 1i) but also on Teff cells (Fig. 1j) and CD11b^+^, Ly6C^low^, F4/80^+^ macrophages (Fig. 1k). Teff cells overexpressed CD39, the other ectonucleotidase involved in ATP degradation (Fig. 1l). Differently, the frequency of myeloid cells, including monocytes/macrophages (CD11b^+^Ly-6C^+^, F4/80^+^, Ly-6G^neg^ gate) and cDC (CD11b^+^, Ly6C^low^, CD11c^+^), is unaffected by the host genetic background (not shown). Data at tumor site are almost mirrored in the spleen of tumor bearer as for Treg increase (Fig. S1A) and overexpression of CD73, OX40, and PD-1 (Fig. S1B–D). This phenotype is tumor-driven because no significant difference in terms of Treg content or CD73, OX40, and PD-1 expression was recorded in spleens of nontumor-bearing controls either WT or P2X7 null (Fig. S1E–H). In accordance with the general immunosuppressive microenvironment of tumors growing in P2X7 null mice, their systemic cytokine levels shifted versus an anti-inflammatory phenotype. Indeed, IL-1β, TNF-α, and IFN-γ levels decreased in tumor-bearing *p2x7*^-/-^ mice whereas TGF-β increased (Fig. 1m–p), confirming the hypothesis that P2X7 deficiency favors immune suppression.

### P2X7 antagonist increases tumor-infiltrating T lymphocytes with reduced expression of CD39 and CD73

**Fig. 2:**
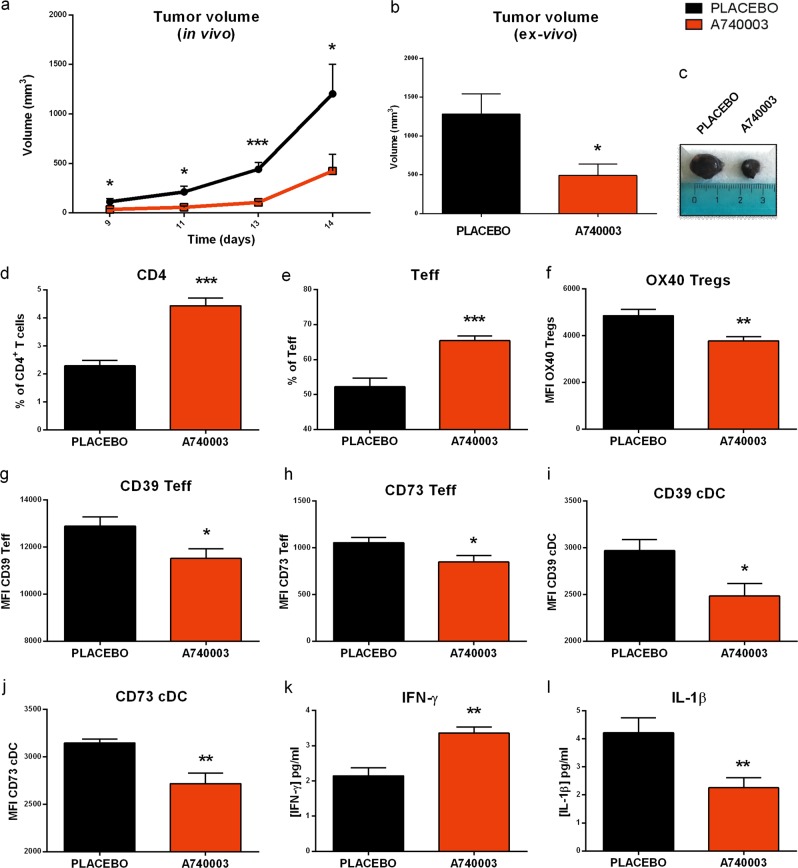
P2X7 antagonism increases tumor-infiltrating T effector lymphocytes and decreases ectonucleotidases expression on T effector and dendritic cells. **a–c** C57bl/6 mice were inoculated into the right hind flank with B16-pmeLUC in WT mice. A740003 (50 µg/kg) was intra-peritoneum administered to mice at post-inoculum days 5, 7, 9, 11, and 13. **a** Tumor volume was in vivo assessed at the indicated time points, **b** ex vivo tumor volume assessed by a calliper, **c** representative pictures of tumors from treated mice at post-inoculum day 14. Data are shown as the mean ± SEM (*n* = 9 per group). **d–j** Flow cytometric analysis of tumor masses from C57bl/6 WT mice treated with placebo or A740003 (50 µg/kg). **d** Percentage of CD4^+-^infiltrating T cells (CD45^+^T gate), **e** percentage of Teff (CD25^-^, Foxp3, CD4^+^ gate), **f** mean fluorescence intensity (MFI) of OX40 on Tregs, **g, h** MFI of CD39 (**g**) and CD73 (**h**) on Teff, **i, j** MFI of CD39 (**i**) and CD73 (**j**) on cDC (cDC, Cd11b^+^ Ly6C^low^, Cd11c^+^ gate). Data are shown as the mean ± SEM. (placebo, *n* = 6–8; A740003, *n* = 4–8). **k, l** P2X7 antagonism increases circulating levels of IFN-γ while decreasing IL-1β. IFN-γ (*n* = 6 per group) (**k**) and IL-1β (**l**) (*n* = 12 per group) were evaluated in plasma samples obtained at post-inoculum day 14. Data are shown as the mean ± SEM. *P < 0.05, **P < 0.01, and ***P < 0.001

Aiming at characterizing the immune cell composition of tumors treated with a P2X7 blocking agent, B16 bearing mice were injected intraperitoneally with the P2X7 antagonist A740003. This treatment reduces tumor growth (Fig. 2a–c) while reshaping the immune infiltrate (Fig. 2d–j). The tumor content of CD4^+^ and Teff lymphocytes significantly increased (Fig. 2d, e) while the frequency of cytotoxic CD8^+^ lymphocytes and Tregs remained unaltered (not shown). Nevertheless, OX40 expression on Treg cells is reduced (Fig. 2f), suggesting a decreased immunosuppressive activity for these cells. Neither CD73 nor CD39 expression is altered on Tregs (not shown), but both ectonucleotidases are significantly down-modulated on Teff and cDC (Fig. 2g–j). As per systemic effect, A740003 administration caused no changes in CD4^+^, Teff, and cDC spleen content, or in spleen Treg CD73 and CD39 expression (Fig. S2). The only alterations in spleen immune cell composition were caused, as expected, by the presence of the growing tumor (Fig. S2). Finally, P2X7 blockade increased systemic levels of IFN-γ (Fig. 2k) and decreased IL-1β (Fig. 2l).

### P2X7 genetic deletion leads to a decrease in TME ATP levels

**Fig. 3:**
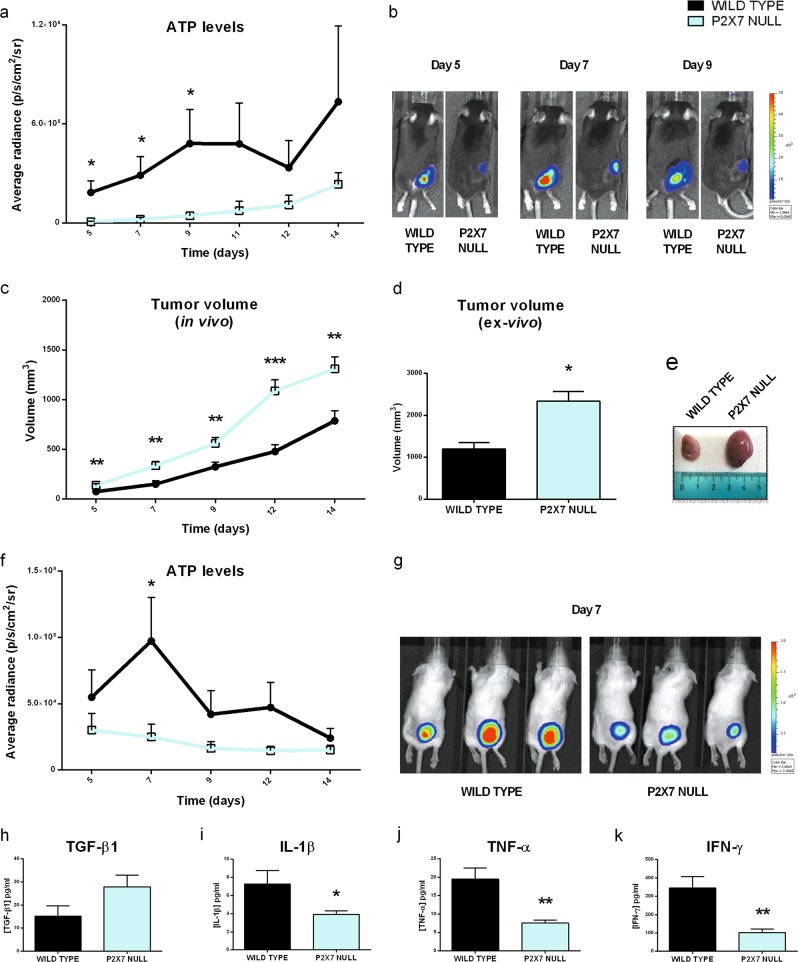
P2X7 ablation leads to a decrease in tumor ATP levels. **a–g** C57bl/6 (**a, b**) and BALBc/J (**c–g**) mice were inoculated into the right hind flank with B16-pmeLUC or WEHI-3B-pmeLUC cells, respectively in WT and P2X7 null mice. **a, f** Measure of ATP levels in tumor-bearing mice estimated by pmeLUC luminescence emission (p/s/cm^2^/sr), **b, g** representative pictures of pmeLUC luminescence emission in C57bl/6 (**b**) tumor-bearing mice at post-inoculum days 5, 7, and 9 and in BALBc/J (**g**) tumor-bearing mice at post-inoculum day 7, **c** tumor volume was in vivo assessed at the indicated time points, **d** ex vivo tumor volume assessed by a calliper, **e** representative pictures of tumors from WT and P2X7 null mice at post-inoculum day 14. Data are shown as the mean ± SEM (C57bl/6 WT, *n* = 6–12; C57bl/6 P2X7 null, *n* = 5–12; BALBc/J *n* = 12 per group). **h–k** Levels of plasma cytokines of tumor-bearing BALBc/J mice inoculated with WEHI-3B-pmeLUC cells. TGF-β1 (**h**) (*n* = 9 per group), IL-1β (**i**) (*n* = 17 per group), TNF-α (**j**) (*n* = 10 per group), and IFN-γ (**k**) (WT, *n* = 4; P2X7 null, *n* = 5) were evaluated in plasma samples obtained at post-inoculum day 14. Data are shown as the mean ± SEM. *P < 0.05, **P < 0.01, and ***P < 0.001

Changes in immune cell ectonucleotidase expression suggest that TME ATP levels might be affected by P2X7 genetic deletion or pharmacological blockade. Using the B16 melanoma cells stably expressing the ATP reporter pmeLUC implanted into P2X7 WT or null mice, we showed that tumor growth in *p2x7*^*−/−*^ mice (Fig. 1a–c) is accompanied by a strikingly reduced quantity of ATP, especially at days 5, 7, and 9 following cancer cell injection (Fig. 3a, b). Similar data were obtained with another P2X7-expressing tumor cell line, i.e. the WEHI-3B murine leukemia cells [13], implanted in the syngeneic BALBc/J host [26, 31] (Fig. 3c–k). WEHI-3B tumor growth is accelerated in *p2x7*^*−/−*^ mice (Fig. 3c–e), and TME ATP levels decreased (Fig. 3f, g). Also varied were the circulating levels of TGF-β that tended to increase (Fig. 3h) and those of proinflammatory cytokines that significantly diminished (Fig. 3i–k). P2X7 pore formation and ATP release have been associated with pannexin1 (panx1) cleavage and opening [32]; therefore, we investigated ATP release in B16 melanoma-bearing panx1^−/−^ mice. No difference was found in TME ATP content between panx1^−^^/−^ and WT mice, suggesting that panx1 does not participate in setting TME ATP levels in this tumor model (Fig. S3).

### Immune cells release ATP depending upon P2X7 expression

**Fig. 4:**
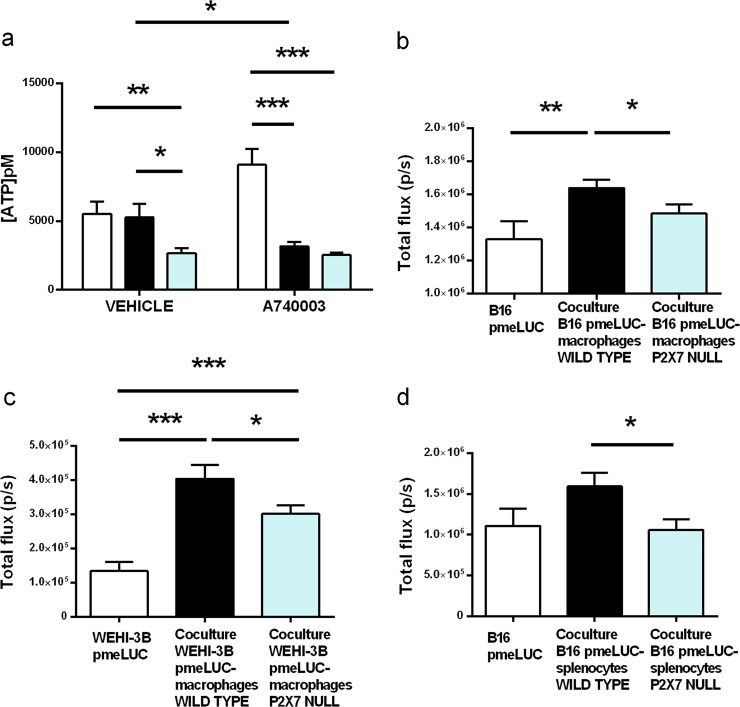
P2X7 null immune cells, alone or in co-colture with cancer cells, show lowered ATP release. **a** Supernatant ATP assessed in B16 and peritoneal macrophages coltures from WT and P2X7 null mice treated with either vehicle (PBS + DMSO 0,1%) or A740003 20 µM. Data are shown as the mean ± SEM (Vehicle, *n* = 7; A740003, *n* = 8). **b, c** Peri-cellular ATP levels in B16-pmeLUC cells (**b**) or WEHI-3B-pmeLUC cells (**c**) alone and in co-culture with peritoneal macrophages derived from C57bl/6 (B16-pmeLUC *n* = 17; Co-culture WT *n* = 34; Co-culture P2X7 null *n* = 25) or BALBc/J (WEHI-3B-pmeLUC *n* = 6; Co-culture WT *n* = 6; Co-culture P2X7 null *n* = 9) WT and P2X7 null mice, respectively. Data are shown as the mean ± SEM. **d** Peri-cellular ATP levels in B16-pmeLUC cells alone or in co-culture with splenocytes derived from C57bl/6 WT and P2X7 null mice. Data are shown as the mean ± SEM (B16-pmeLUC *n* = 8; Co-culture WT *n* = 13; Co-culture P2X7 null *n* = 15). Luminescence data were expressed as total photons acquired in a 5 min session. *P < 0.05, **P < 0.01, and ***P < 0.001

In an effort to identify the cellular source of TME ATP, we measured spontaneous ATP release from tumor cells or peritoneal macrophages isolated from either P2X7 WT or P2X7 null mice (Fig. 4a). ATP release from P2X7 null was almost halved compared to P2X7 WT macrophages and a similar effect was obtained upon A740003 administration (Fig. 4a). Interestingly, P2X7 blockade also caused a rise in ATP released by B16 cells (Fig. 4a). Co-culture of macrophages with B16 or WEHI-3B pmeLUC cells promoted an increase in ATP release, that was, however, lower in the presence of P2X7 null macrophages (Fig. 4b, c). Similar results were retrieved when co-culturing B16-pmeLUC cells with either WT or P2X7 null splenocytes (Fig. 4d), suggesting that the lower level of ATP in P2X7 null mice could mainly depend on a decreased release by host immune cells.

### P2X7 pharmacological blockade has no effect on TME ATP but increases ATP release from tumor cells in vitro

**Fig. 5:**
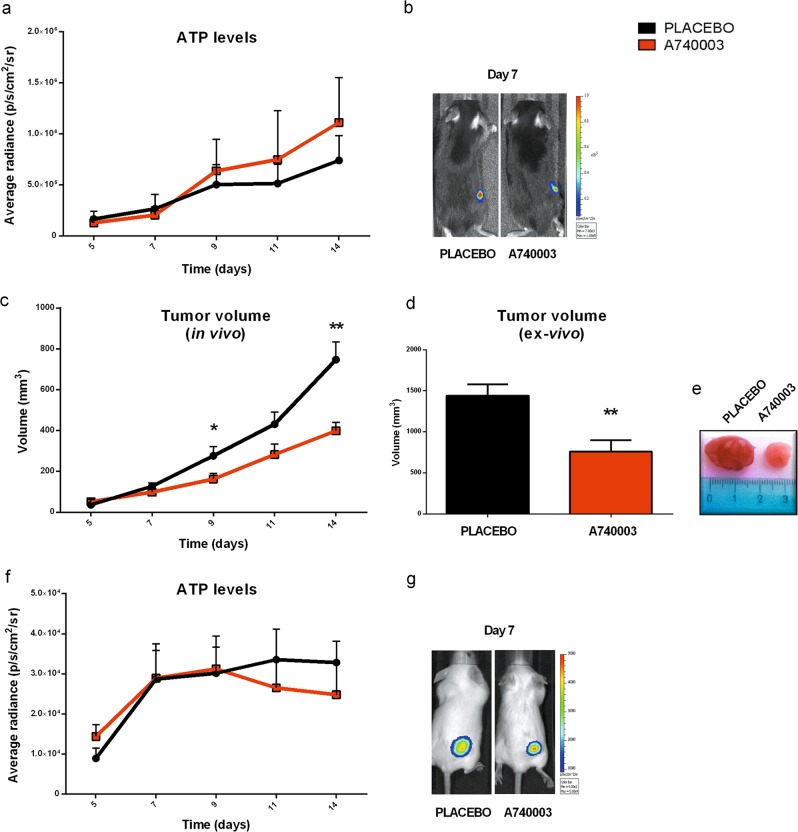
P2X7 antagonist systemic administration does not influence ATP levels in vivo. C57bl/6 (**a, b**) and BALBc/J (**c–g**) mice were inoculated into the right hind flank with B16-pmeLUC or WEHI-3B-pmeLUC cells, respectively, in WT mice. A740003 (50 µg/kg) was intra-peritoneum administered to mice at post-inoculum days 5, 7, 9, 11, and 13. **a**, **f** Measure of ATP levels in treated mice estimated by pmeLUC luminescence emission (p/s/cm^2^/sr), **b**, **g** representative picture of pmeLUC luminescence emission in mice at post-inoculum day 7, **c** tumor volume was in vivo assessed at the indicated time points, **d** ex vivo tumor volume assessed by a calliper, **e** representative pictures of tumors from treated mice at post-inoculum day 14. Data are shown as the mean ± SEM (*n* = 9 per group). *P < 0.05 and **P < 0.01

**Fig. 6:**
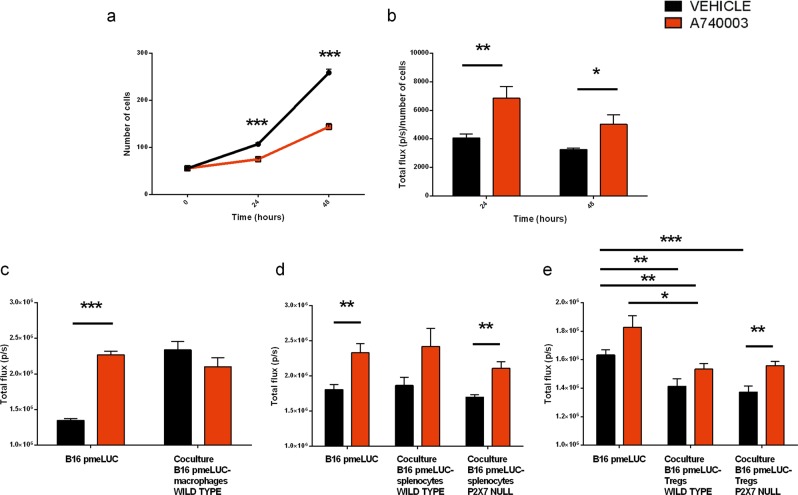
A740003 treatment leads to B16-pmeLUC cell growth arrest accompanied by ATP release. **a** Proliferation at 0, 24, and 48 h of B16-pmeLUC cells treated with vehicle (PBS + 0.1% DMSO) or A740003 20 µM (*n* = 6 per group). **b** ATP levels of B16-pmeLUC cells treated as above described. Luminescence data are normalized on cell number and expressed as total photons measured in a 5 min acquisition (*n* = 6 per group). **c, d** P2X7 antagonism in co-culture of tumor cells and macrophages or splenocytes results in unaltered ATP levels. **c** ATP levels in B16-pmeLUC cells alone and in co-culture with peritoneal macrophages derived from C57bl/6 WT mice treated with vehicle (PBS + 0.1% DMSO) or A740003 20 µM (Vehicle, *n* = 4; A740003, *n* = 6), **d** ATP levels in B16-pmeLUC cells alone and in co-culture with splenocytes derived from C57bl/6 WT and P2X7 null mice treated with vehicle (PBS + 0.1% DMSO) or A740003 20 µM (Vehicle, *n* = 6–10; A740003, *n* = 9–12). **e** Tregs reduce ATP secretion from cancer cells. ATP levels in B16-pmeLUC cells alone and in co-culture with Treg cells derived from C57bl/6 WT and P2X7 null mice treated with vehicle (PBS + 0.1% DMSO) or A740003 20 µM (*n* = 9 per group). **c–e** Luminescence data are expressed as total photons measured in a 5 min acquisition. Data are shown as the mean ± SEM. *P < 0.05, **P < 0.01, and ***P < 0.001

A740003 administration while significantly reducing tumor growth did not affect ATP concentration in the TME of mice implanted with B16 or WEHI-3B cells (Fig. 5a, b, f, g). Since ATP release is decreased by immune cell P2X7 blockade but is increased by P2X7 antagonism on tumor cells (see Fig. 4a), we further investigated the effect of P2X7 blockade in B16 and WEHI-3B in vitro alone or in co-colture with host immune cells. As shown in Fig. 6, A740003 caused a significant inhibition of B16 proliferation (Fig. 6a), paralleled by increased ATP release (Fig. 6b). A similar effect on cell growth and ATP release is observed in WEHI-3B cells and by treatment with another P2X7 blocker, already proved to reduce B16 growth in vivo [22, 26]: AZ10606120 (Fig. S4A-F). However, the A740003-stimulated increase in ATP release was lost in tumor cells and WT macrophages or splenocytes co-cultures (Fig. 6c, d), possibly due to a balance between increased tumor cells and decreased immune cells ATP release (see also Fig. 6a). Accordingly, in co-cultures of B16 cells with P2X7 null splenocytes, A740003 significantly increased peri-cellular ATP replicating the effect of the drug on isolated B16 in the absence of host P2X7 (Fig. 6d). Due to the reported overexpression of CD73 on P2X7 null Tregs (Fig. 1i), we tested whether there is a direct contribution of Tregs to ATP down-modulation. Co-culturing Tregs, isolated from either P2X7 WT or null spleen, with B16 cells we measured a significant decrease in peri-cellular ATP concentration (Fig. 6e). Interestingly, administration of A740003 partially reverted this effect by increasing ATP (Fig. 6e).

### P2X7 blockade reduces tumor cell growth while increasing CD4^+^ infiltrate and lowering CD73 expression in P2X7 null mice

**Fig. 7:**
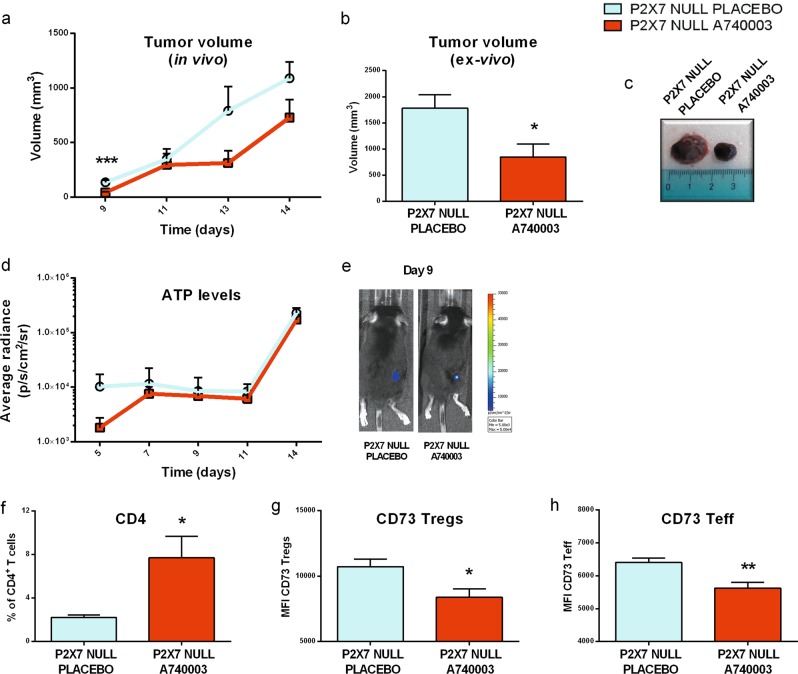
P2X7 blockade reduces tumor growth in P2X7 null mice. **a–e** C57bl/6 mice were inoculated into the right hind flank with B16-pmeLUC in P2X7 null mice. A740003 (50 µg/kg) was intra-peritoneum administered to mice at post-inoculum days 5, 7, 9, 11, and 13. **a** Tumor volume was in vivo assessed at the indicated time points, **b** ex vivo tumor volume assessed by a calliper, **c** representative pictures of tumors from treated mice at post-inoculum day 14, **d** kinetics of ATP levels in treated mice estimated by pmeLUC luminescence emission (p/s/cm^2^/sr), **e** representative picture of pmeLUC luminescence emission in mice at post-inoculum day 9 (placebo, *n* = 7; A740003, *n* = 8). **f–h** Ex vivo characterization of T cells by cytometric analysis in tumor masses derived from C57bl/6 P2X7 null mice treated with placebo or A740003 (50 µg/kg). **f** Percentage of CD4^+^ infiltrating T cells (*n* = 5 per group), **g**, **h** MFI of CD73 on Tregs (**g**) (placebo, *n* = 5; A740003, *n* = 7) and Teff (**h**) (placebo, *n* = 5; A740003, *n* = 6). Data are shown as the mean ± SEM. *P < 0.05, **P < 0.01, and ***P < 0.001

In an effort to analyze in vivo the effect of selective tumor-P2X7 blockade, we investigated the impact of P2X7 antagonism on P2X7 null mice implanted with P2X7-expressing B16 tumors. Treatment with A740003 significantly reduced B16 growth also in P2X7 null mice (Fig. 7a–c) but did not alter ATP levels in TME (Fig. 7d, e). Interestingly, the tumor infiltrate of P2X7 null bearing mice treated with A740003 was characterized by increased CD4^+^ levels (Fig. 7f) and decreased expression of CD73 on both Treg and Teff cells (Fig. 7g, h), possibly due to factors released by tumor cells following P2X7 blockade. No other tested tumor-infiltrating populations or their CD73 and CD39 expression was altered in this model (not shown).

## Discussion

**Fig. 8:**
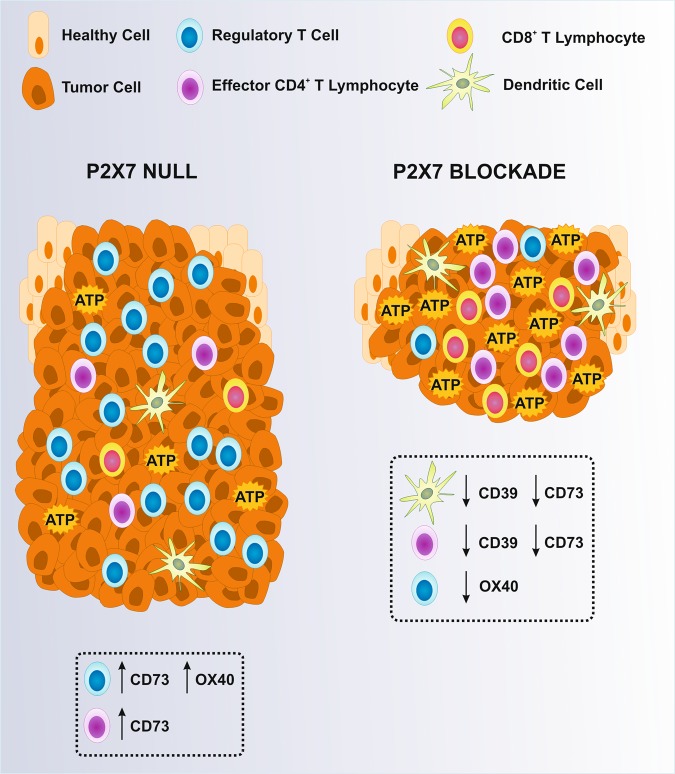
Schematic representation of the main findings of the study. Tumors growing in P2X7 null host show an increase of volume accompanied by a generation of an immunosuppressive microenvironment, characterized by a decrease of ATP levels and an augmented number of regulatory T cells overexpressing OX40 and CD73. P2X7 pharmacological blockade leads to a reduction of tumor volume and an increase of tumor-infiltrating T lymphocytes that express low levels of CD39 and CD73. Tregs and conventional dendritic cells while remaining unaltered in numbers down-modulate, respectively, OX40, CD39 and CD73

The P2X7 receptor for ATP is a promising therapeutic target in oncology owing to the efficacy of P2X7 antagonists in reducing tumor growth and metastatic spread in preclinical models [4]. However, both ATP increase in the TME and P2X7 engagement on immune cells were also associated with anti-tumoral immune response [33, 34]. The contrasting roles played by host versus cancer cell P2X7 clearly emerged in recent studies demonstrating that host P2X7 deficiency increased cancer progression [26, 29]. Here we further analyzed the immune contexture of tumors growing in P2X7 null mice as opposed to those exposed to P2X7 antagonists. Tumor infiltrate analysis in P2X7 null versus A740003 treated mice revealed strong differences in T cells populations. Indeed, while tumors implanted in P2X7 null host are characterized by a strong reduction in CD8^+^ and Teff cells and an increased frequency in immunosuppressive Tregs (present study and see [35−38]), P2X7 antagonism causes a rise of Teffs while leaving unaltered CD8^+^ and Tregs numbers. Via down-modulation of OX40, P2X7 antagonism also reduces Tregs immunosuppressive performance [39]. The systemic effects of tumors implanted in P2X7 null versus P2X7 antagonized hosts are likewise different. Increased Tregs frequency and expression of OX40 and PD-1 is found also in the spleen population of P2X7 null host. On the contrary, A740003 administration did not alter spleen immune cells composition, either in the presence nor in the absence of tumor. These data, while not totally excluding off-target effects of A740003, suggest a general lack of immune side-effects consequent to P2X7 blockade. In both melanoma and leukemia models tested, the circulating levels of cytokines shifted versus an immune suppressive phenotype [40, 41], while P2X7 antagonism caused an increase in IFN-γ levels. Analysis of ectonucleotidases expression on tumor-infiltrating immune cells allowed us to correlate P2X7 with the adenosine-generating pathway, which attracted great attention for its role in tumor-immune suppression [2]. This pathway includes CD39, which is responsible for the conversion of ATP into ADP and AMP, and CD73, which hydrolyzes AMP into adenosine and represents the rate-limiting step for the formation of the nucleoside [42]. Interestingly, P2X7 null mice upregulate CD73 on tumor-infiltrating Tregs, further supporting an improved immunosuppressive performance of these immune cells [34, 35], and thus demonstrating an inverse correlation among P2X7 and CD73 expression. CD73 expression is enhanced on tumor-infiltrating monocyte/macrophages and Teffs, which upregulate also CD39, suggesting a general rise in adenosine at the expenses of ATP in the TME of P2X7 null mice possibly causing a functional compensation in these genetically modified animals. Moreover, these data are in accordance with the opposing immune-suppressing versus immune-promoting functions of CD39 and P2X7 [43–47]. Tumor-bearing mice treated with the P2X7 antagonist A740003 differ from *p2x7*^−/−^ hosts also in the expression of ectonucleotidases. Indeed, CD39 and CD73 are down-modulated on Teff and dendritic cells, suggesting that P2X7 blockade will be reducing ATP degradation into adenosine. Data on ectonucleotidases and the recognized role of P2X7 as ATP conduit [7, 48] prompted us to investigate ATP levels in the TME of our experimental models. We demonstrated that the ATP levels in the TME of P2X7 null mice is lowered, while receptor’s blockade did not lower ATP levels in the TME of P2X7 WT mice. A reduction in ATP release in P2X7 null mice was recently demonstrated in another pathologic model: allograft rejection [10] suggesting that this could be a general characteristic of *p2x7*^−^^/−^ mice during immune reactions. Therefore, aiming at identifying the source of TME ATP, we measured ATP release from immune and cancer cells alone or in co-culture. P2X7 null macrophages secrete reduced amounts of ATP as compared to WT and a similar effect is obtained by treating WT macrophages with A740003. However, this P2X7 antagonist causes also a striking increase in ATP release from B16 and WEHI-3B cancerous cells that was reproducible upon administration of another, structurally unrelated, P2X7 blocking drug: AZ10606120. These data suggest that following antagonism of P2X7, tumor cells will release ATP activating an anti-tumoral immune response. Co-culture of the same tumor cells with WT macrophages or splenocytes results in a significant increase in peri-cellular ATP that is almost lost in the presence of *p2x7*^−/−^ immune cells, while remains unaltered following antagonist administration. Finally, Tregs are the only immune cells that in co-culture reduce cancer cells' plasma membrane ATP. Taken together our data suggest that, in P2X7 null mice, the decrease of TME’s ATP will be mainly ascribable to a defect in host immune cells release and an increase in ATP degradation mediated by infiltrating Tregs. On the contrary, treating with P2X7 antagonists will, on the one hand, increase ATP release from tumor cells, but will on the other decrease it from immune cells, thus leaving unaltered the overall content of ATP in the TME. Finally, we tested the effect of P2X7 blockade on *p2x7*^−/−^ hosts bearing tumors that express the P2X7 receptor as they derive from implanted B16 cells. Also in this model A740003 treatment caused a significant reduction in tumor growth similar, if not increased, to the WT host and possibly ascribable to a rise in CD4^+^. These data give a further confirmation that P2X7 blockade exerts its anti-tumoral activity mainly reducing P2X7-dependent tumor cell growth. In conclusion, we demonstrated that P2X7 receptor is able to influence the entire TME purinergic signaling system modulating the secretion of ATP from both immune and tumor cells, but also acting via its degradation through ectonucleotidases expressed from different immune populations including Tregs, Teff, and cDC (Fig. 8). Increased expression of the adenosine-generating enzyme CD73 in P2X7 null mice is mirrored by a decrease in ATP concentration in the TME, a condition that blunts immunostimulation in favor of immunosuppression. On the contrary, ATP levels remain unaltered in the TME of mice treated with the P2X7 antagonist, due to increased release of ATP from tumor cells and decreased ectonucleotidase expression, with a net result of a robust tumor inhibition owing to combined immune effect and direct action on tumor cells’ P2X7. Finally, our data strongly suggest caution in extending findings obtained in P2X7 null mice to the therapeutical application of P2X7 targeting compounds.

## Materials and methods

### Cell cultures and generation of pmeLUC expressing stable clones

B16 melanoma and WEHI-3B myelomonocytic leukemia macrophage-like cells were previously available at Drs. Adinolfi and Curti laboratories [13, 22, 26] and resulted mycoplasma free by mycoAlert detection kit (Lonza, distributed by Euroclone, Milan; Italy) testing. B16 and WEHI-3B cells were grown, respectively, in RPMI-1640 medium (Sigma-Aldrich, Saint Louis, Missouri, USA) plus non-essential amino acids (Sigma-Aldrich) and in Iscove’s Modified Dulbecco’s Medium (Sigma-Aldrich), both supplemented with 10% fetal bovine serum (FBS) (Euroclone, Milan,Italy), 100 U/ml penicillin (Euroclone), and 100 mg/ml streptomycin (Euroclone). To obtain B16 and WEHI-3B cell clones stably expressing plasma membrane luciferase, cells were transfected with the pmeLUC probe, as previously described [13, 49]. B16 was transfected with Lipofectamine LTX (Thermo Fisher Scientific, Waltham, Massachusetts, USA), as per the manufacturer's instructions, while WEHI-3B were electroporated as follows: 6 × 10^6^ cells were suspended in electroporation buffer (Thermo Fisher Scientific) in the presence of 3 μg of plasmid’s DNA and placed in a Microporator MP-100 (Thermo Fisher Scientific), applying a voltage of 1250 V for 40 ms. Stably transfected cell clones were obtained by selection with neomycin/G418 sulfate (0.2–0.8 mg/mL; Sigma-Aldrich) followed by limiting dilution as previously described [49].

### Mice strains, tumor generation, in vivo imaging, and drug administration

In vivo experiments were performed with two different *p2x7*^*−/−*^ mouse strains and corresponding WT controls: C57bl/6, a gift from GlaxoSmithKline to F Di Virgilio and BALBc/J kindly provided by N R Jørgensen, University Hospital Glostrup, Glostrup, Denmark [26]; or *panx1*^*−/−*^ mice in the C57bl/6 strain, kindly supplied by H Monyer, Department of Clinical Neurobiology, University Hospital of Neurology, Heidelberg, Germany [50]. Based on calculations performed with the G-power software [51] on previously published data [26], a sample size of nine animals per group was chosen to achieve a predicted power of 0.9 with an effect size of .45 using a two-tailed *t*-test. A total of 2.5 × 10^5^ B16-pmeLUC or 2 × 10^6^ WEHI-3B-pmeLUC cells were subcutaneously injected into C57bl/6 or BALBc/J 4–6 weeks old female mice, respectively. Tumor size was measured with a calliper, and volume calculated according to the following equation: volume = *π*/6 [*w*_1_ × (*w*_2_)^2^], were *w*_1_ is the major diameter and *w*_2_ is the minor diameter. Luminescence emission was measured daily, day 5 to 14 from tumor cell inoculum with a total body luminometer for small animals (IVIS Lumina, Caliper; Perkin Elmer, Hopkinton, Massachusetts, USA). Mice anesthetized with 2.5% isofluorane were intra-peritoneum injected with 150 mg/kg d-luciferin (Promega, Madison, Wisconsin, USA) and, after a 15-min interval allowing for biodistribution, luminescence was captured from dorsal view. Photon emission was quantified using the Living Image® software (Perkin Elmer) and averaged as photons/seconds/cm^2^/steradian (abbreviated as p/s/cm^2^/s). P2X7 antagonist A740003 (Tocris Bioscience, Bristol, UK) or vehicle (sterile phosphate-buffered saline (PBS), 0.005% DMSO) were intra-peritoneum injected (100 μl) every 2 days after day 5, corresponding, when applicable, to first tumor mass detection. Mice were randomized in groups with the free software research randomizer (www.randomizer.org) and the operator was blinded to the group of allocation during the experiment. The administered concentration (50 μg/kg) of A740003 was in line with those shown to reduce experimental cancer growth [24, 26] and neuropathic pain [52]. A740003 shows good half-life and biodistribution in vivo [52, 53]. Blood samples were collected from the submandibular vein under general anesthesia immediately before sacrificing the animal (post-inoculum day 14). Tumors were excised and processed for further analysis. Mouse plasma was collected by centrifugation (1000 × *g*, 10 min at 4 °C) of blood and stored at −80 °C in the presence of Halt Protease and Phosphatase Inhibitor Cocktail, EDTA-Free (Thermo Fisher Scientific). All animal procedures were approved by the University of Ferrara Ethic committee and the Italian Ministry of Health in compliance with international laws and policies (EU Directive 2010/63/EU and Italian D.Lgs 26/2014).

### Evaluation of the tumor-immune infiltrate by flow cytometry

Tumor-infiltrating lymphocytes and myeloid cells were characterized on tumor cell suspension after separation by Ficoll gradient (GE Healthcare Life Sciences, Little Chalfont, Buckinghamshire, UK). For the staining of surface markers, tumor-infiltrating lymphocytes were incubated for 30 min at 4 °C in the dark with the following anti-mouse mAbs: CD45 BV605 (30-F11; BD-Biosciences, Milan, Italy); CD3 PE-Cy7 (clone 17A2; BioLegend, San Diego, CA, USA), CD4 BV510 (BioLegend) or APC-Cy7 (HL3; BD-Biosciences), OX40-PE (clone OX86; BioLegend), PD-1 BV711 (29F.1A12; BioLegend), CD8a PE-Cy7 (53-6.7; BioLegend), TIM3-BV421 (clone RMT3-23; BioLegend) CD25-FITC (PC61; BioLegend), CD39-PE (clone DUHA59; BioLegend) and CD73-BV605 (clone 7Y/11.8; BioLegend) according to the manufacturer’s instruction. For intracellular staining of Foxp3 APC (FJK-16S; Thermo Fisher Scientific) cells were fixed and permeabilized with “Foxp3/Transcription Factor Staining Buffer Set” (Thermo Fisher Scientific) and then stained for 30 min at 4 °C in the dark. Myeloid cells were characterized using mAb to CD206-Alexa 488 (clone 19; Thermo Fisher Scientific), CD11b-BV711 (clone M1/70; Thermo Fisher Scientific), CD11c-PE-Cy7 (clone HL3; Thermo Fisher Scientific), GR-1-APC (clone RB6-8C5; Thermo Fisher Scientific), Ly6C-BV421 (clone AL21; BD-Biosciences), F4/80-BV786 (clone BM8; Thermo Fisher Scientific). Flow cytometry data were acquired on an LSRFortessa (Becton Dickinson, Franklin Lakes, New Jersey, USA) and analyzed with FlowJo software (version 8.8.7, Tree Star Inc.) from tumor mass by Ficoll (GE Healthcare Life Sciences) gradient.

### Cytokines evaluation

Cytokines levels were evaluated following 1:2 plasma dilution either with Mouse TGF-β1, IL-1β, TNF-α (Boster, distributed by Tema Ricerca, Bologna, Italy), IFN-γ (R&D, Bio-techne) ELISA kits or with Ciraplex CK1 mouse multi-cytokine assay kit (Aushon Biosystem, distributed by Tema Ricerca, Bologna, Italy) as per the manufacturer’s instructions.

### In vitro measure of ATP levels

ATP was measured in the culture supernatants with ENLITEN rLuciferase/Luciferin reagent (Promega), as per the manufacturer’s instructions. Briefly, 50 × 10^3^ B16 cells and peritoneal macrophages per well were plated in six-well plates. Following adhesion cells were incubated for 48 h in the presence of vehicle or A740003 (Tocris Bioscience) 20 µM, corresponding to in vivo administered concentration. Luminescence from cell supernatants was measured following addition of 100 μl of ENLITEN reagent per well, in a Perkin Elmer Wallac Victor3 1420 luminometer (Perkin Elmer, Wellesley, Massachusetts, USA). Peri-cellular ATP levels were also measured, thanks to the pmeLUC probe and the use of an IVIS Lumina luminometer (Caliper). d-Luciferin (Promega) was added to all wells at a concentration of 60 μg/ml. Luminescence data were expressed as total photons measured in a 5 min acquisition.

### Immune cells isolation and co-culture conditions

Macrophages were recovered from WT or *p2x7*^−^^*/−*^ mice by peritoneal lavage as described previously [54]. Briefly, the peritoneal cavity was lavaged with ten 1-ml aliquots of sterile PBS (pH 7.4), and cells were harvested by centrifugation at 200 × *g* at 4 °C for 5 min. Spleens were isolated, homogenized by careful pulping, and treated with red blood cell lysis buffer (Roche, Basel, Switzerland) for 5 min at room temperature to remove erythrocytes. The cell suspension was then supplemented with RPMI-1640, centrifuged for 10 min at 150 × *g*, filtered through a 70 μm cell strainer (Becton Dickinson, Franklin Lakes, NJ, USA), rinsed twice with RPMI-1640, and finally re-suspended in the same medium at a concentration of 1.5 × 10^6^ cells/ml [55]. T regulatory cells were isolated from mice spleens with the “CD4^+^ CD25^+^ regulatory T cell isolation kit” (Macs, Miltenyi Biotec, Germany) as per the manufacturer’s instructions. Macrophages were co-cultured with HLA-matched pmeLUC-expressing tumor cells at the following ratios: 1/1 for B16 cells and 1/3 for WEHI-3B cells. Splenocytes and isolated Tregs were co-cultured with B16-pmeLUC-expressing cells respectively at a 40/1 and 10/1 ratios. Supernatant ATP levels were determined with the ENLITEN kit while tumor cell surrounding ATP was assessed by pmeLUC luminescence measure as above described.

### Cell counts

25 × 10^3^ B16-pmeLUC or 150 × 10^3^ WEHI-3B-pmeLUC cells were plated in six-well plates in complete medium and maintained at 37 °C in a CO_2_ incubator. Cells were counted at various time intervals (0, 24, 48 h after drug treatment) in a Bürker chamber with a phase contrast Olympus microscope (Olympus Life Science Europe, Hamburg, Germany).

### Statistics

All data are shown as mean ± standard error of the mean (SEM). Significance was calculated assuming equal SD and variance, with a two-tailed Student’s *t*-test performed with the GraphPad Prism software (GraphPad, La Jolla, California, USA). Multiple *t*-tests were performed analyzing each row individually, not assuming consistent SD and correcting with the Holm–Sidak method. *P*-values lower than 0.05 were considered statistically significant.

## Supplementary information

Supplemental Information
