# Supplementary material for: The P2X7 receptor modulates immune cells infiltration, ectonucleotidases expression and extracellular ATP levels in the tumor microenvironment
Source: Oncogene. 2019 Jan 17;38(19):3636–50. doi: 10.1038/s41388-019-0684-y (PMC6756114; doi:10.1038/s41388-019-0684-y)
Supplement: Supplementary file 1 — Supplemental Information [file 41388_2019_684_MOESM1_ESM.pdf]

Supplemental Information for

The P2X7 receptor modulates immune cells infiltration, ectonucleotidases expression and extracellular ATP levels in the tumor microenvironment

by

Elena De Marchi, Elisa Orioli, Anna Pegoraro, Sabina Sangaletti, Paola Portararo, Antonio Curti, Mario Paolo Colombo, Francesco Di Virgilio, Elena Adinolfi*

*To whom correspondence should be addressed. E-mail: elena.adinolfi@unife.it

This PDF file includes:

Supplemental Figures S1 to S4 and legends (pages 2 to 6)

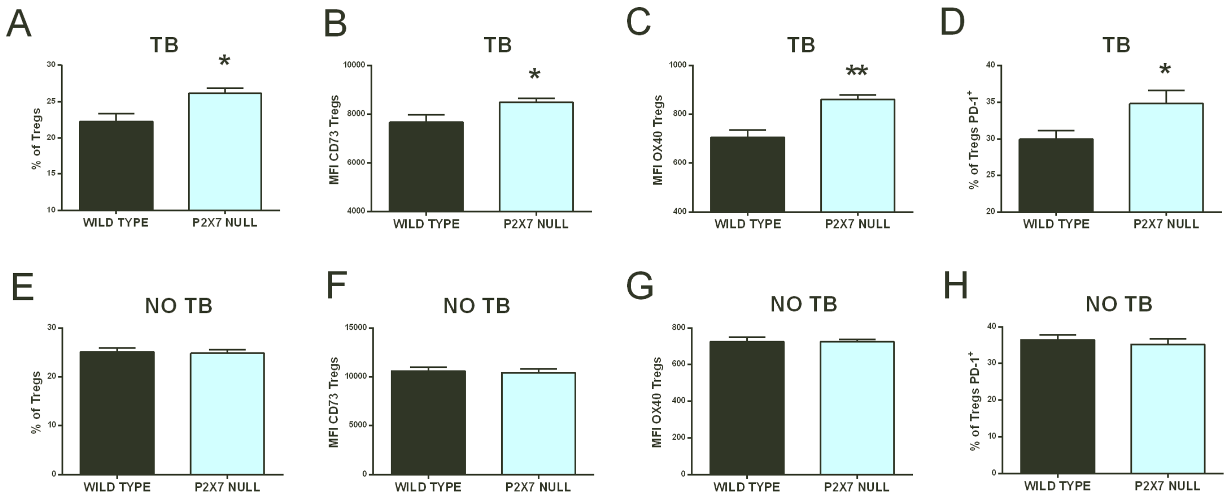

Supplemental Figure S1 Tumor-bearing P2X7 null host shows increased spleen-Tregs. **A-D** Flow cytometric analysis of spleens from tumor bearing C57bl/6 WT and P2X7 null mice. **A** Percentage of Tregs (CD25$^+$, Foxp3$^+$, CD4$^+$ gate), **B** mean fluorescence intensity (MFI) of CD73$^+$ Tregs (Foxp3$^+$ gate), **C** MFI of OX40 on Tregs, **D** percentage of Tregs PD-1$^+$. Data are shown as the mean ± SEM. (n = 6 per group). **E-H** *Ex-vivo* cytometric characterization of spleen-derived T cells from C57bl/6 WT and P2X7 null mice. **E** Percentage of Tregs (CD25$^+$, Foxp3$^+$, CD4$^+$ gate), **F** mean fluorescence intensity (MFI) of CD73$^+$ on Tregs (gated on Foxp3$^+$ cells), **G** MFI of OX40 on Tregs, **H** percentage of PD-1$^+$ Tregs. Data are shown as the mean ± SEM. (n = 5-6 per group).

*P<0.05, **P<0.01 and ***P<0.001.

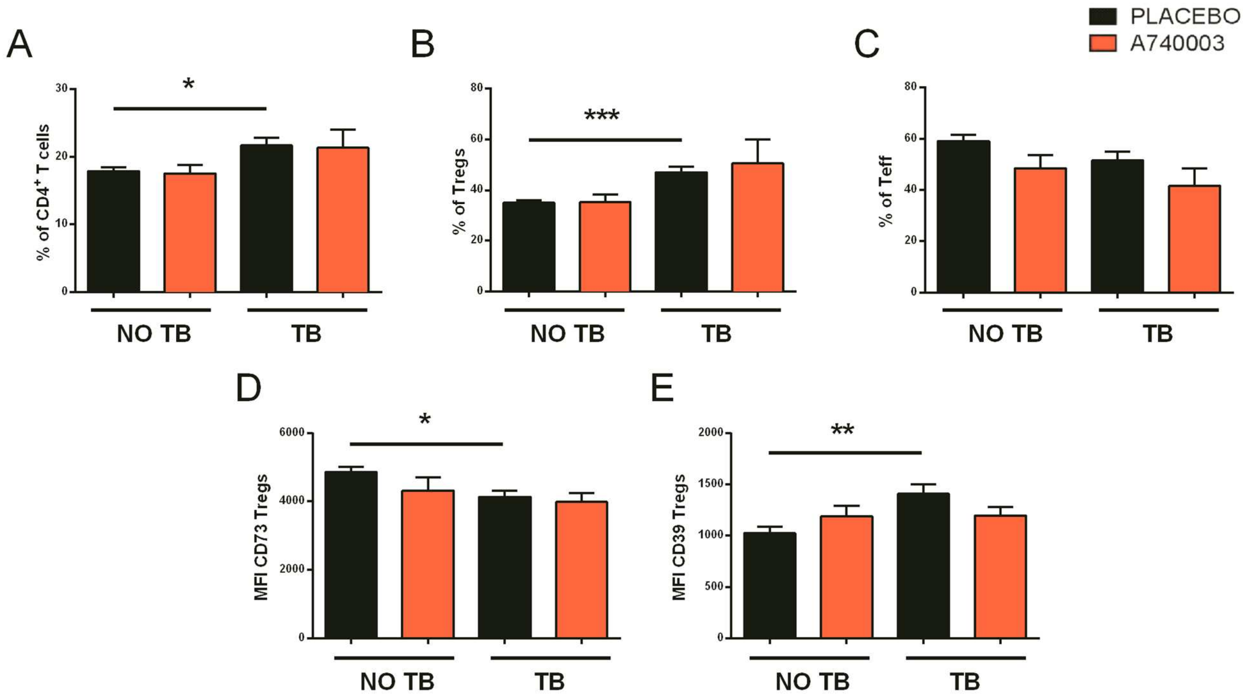

Supplemental Figure S2 A740003 administration does not alter spleen immune cells content. A-E *Ex-vivo* cytometric characterization of spleen-derived T cells from tumor-bearing and not tumor-bearing C57bl/6 WT mice treated with placebo (PBS+0,1% DMSO) or A740003 (50 µg/kg). **A** Percentage of $CD4^+$ T cells ($CD45^+$ gate), **B** percentage of Tregs ($CD25^+$, $Foxp3^+$, $CD4^+$ gate), **C** percentage of Teff ($CD25^-$, $Foxp3^-$, $CD4^+$ gate), **D, E** MFI of CD73 (**D**) and CD39 (**E**) on Tregs. Data are shown as the mean ± SEM. (Placebo NO TB, n = 5; A740003 NO TB, n = 5; Placebo TB, n = 5; A740003 TB, n = 4).

*P<0.05, **P<0.01 and ***P<0.001.

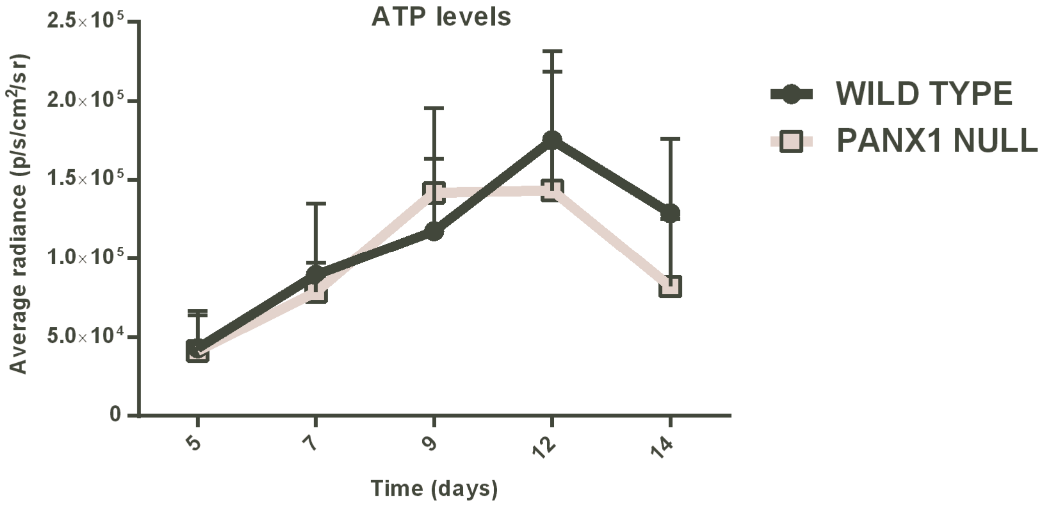

Supplemental Figure S3 ATP levels are not altered in panx1 null mice compared to WT. C57bl/6 mice were inoculated into the right hind flank with B16-pmeLUC in WT and panx1 null mice. ATP levels in mice estimated by pmeLUC luminescence emission (p/s/cm^2/sr). Data are shown as the mean ± SEM. (n = 6 per group).

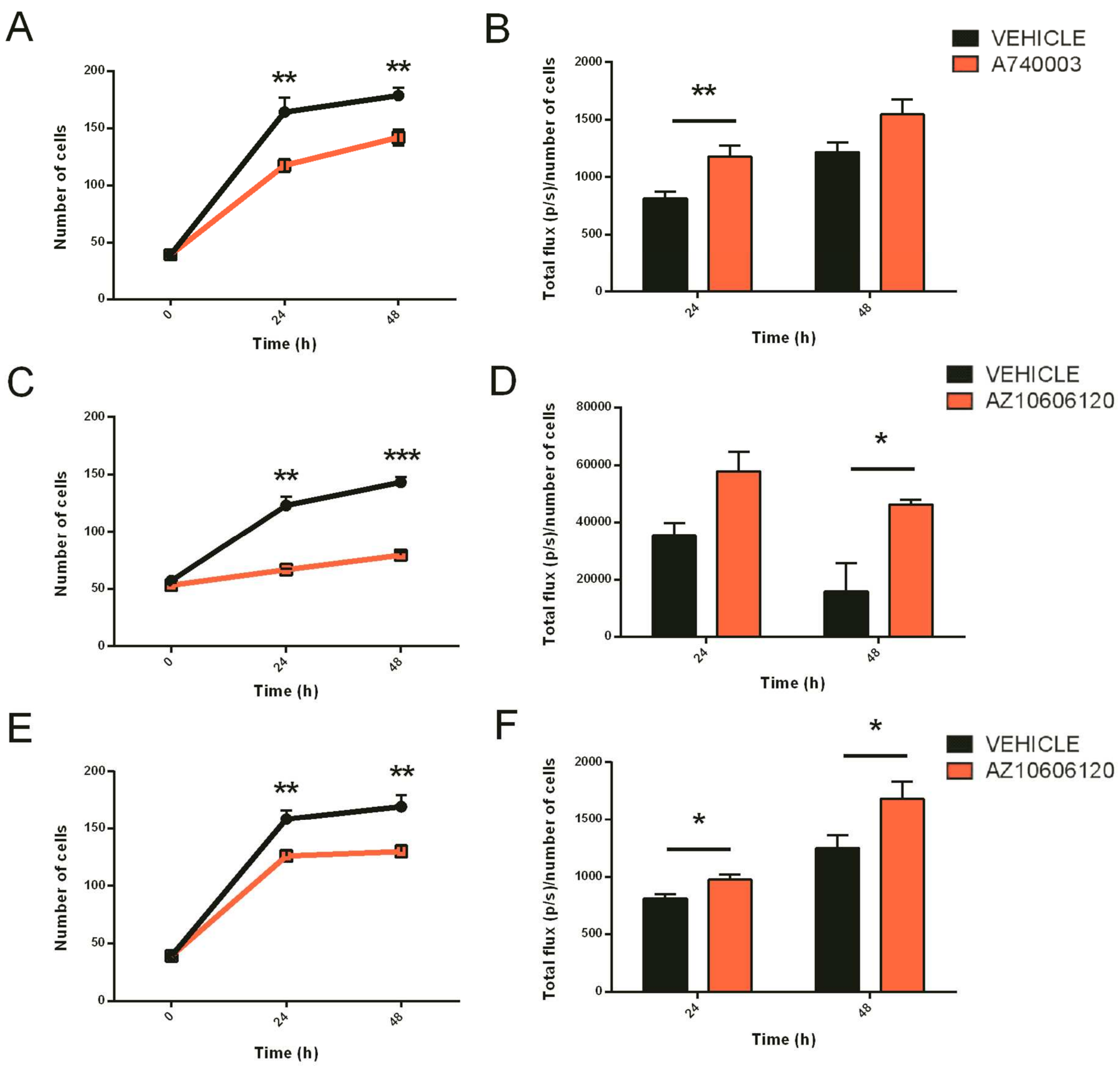

Supplemental Figure S4 P2X7 antagonism leads to B16-pmeLUC or WEHI-3B-pmeLUC cell growth arrest accompanied by ATP release. (**A**) Proliferation at 0, 24 and 48 hours of WEHI-3B-pmeLUC cells treated with vehicle (PBS+0,1% DMSO) or A740003 20µM. Data are shown as the mean ± SEM. (n = 6 per group). (**B**) ATP levels of WEHI-3B-pmeLUC cells treated as above described. Data are shown as the mean ± SEM. (n = 6 per group). **C, E** Proliferation at 0, 24 and 48 hours of B16 (**C**) (n = 3 per group) or WEHI-3B (**E**) (n = 6 per group) pmeLUC cells treated with vehicle (PBS) or AZ10606120 1µM. Data are shown as the mean ± SEM. **D, F** ATP levels of B16 (**D**) (n = 3 per group) or WEHI-3B (**F**) (n = 6 per group)

pmeLUC cells treated as above described. Data are shown as the mean ± SEM. Luminescence data are expressed as total photons measured in a 5 minutes acquisition. *$P<0.05$, **$P<0.01$ and ***$P<0.001$.
